# Retraction: Evaluating the efficacy, safety, and immunogenicity of FDA-approved RSV vaccines: a systematic review of Arexvy, Abrysvo, and mResvia

**DOI:** 10.3389/fimmu.2026.2009934

**Published:** 2026-10-02

**Authors:** Frontiers Editorial Office

The journal retracts the 18 August 2025 article cited above.

Following publication, concerns were raised regarding the bibliographic references in the published article. The authors failed to provide a satisfactory explanation during the investigation, which was conducted in accordance with Frontiers’ policies. As a result, the data and conclusions of the article have been deemed unreliable, and the article has been retracted.

This retraction was approved by the Chief Editors of Frontiers in Immunology and the Chief Executive Editor of Frontiers. The authors were informed regarding this retraction.

